# Supplementary material for: Complexome profiling of the Chlamydomonas psb28 mutant reveals TEF5 as an early PSII assembly factor
Source: Plant Cell. 2025 Mar 17;37(6):koaf055. doi: 10.1093/plcell/koaf055 (PMC12164590; doi:10.1093/plcell/koaf055)
Supplement: koaf055_Supplementary_Data [file koaf055_supplementary_data.zip › psb28_interactive.zip!/plots/intensity/qualityPlots/peptides/RPS25_Cre08.g382500.t1.2.html]

### Cre08.g382500.t1.2

Trivial name: RPS25  
SeqCoverage [median]: 26.20  
Mass: 11.82 kDa  
  
 

| Sequence | MissedCleavage | Mass [Da] | wtIntensity | muIntensity | Score |
| --- | --- | --- | --- | --- | --- |
| ALAAANSSK | 0 | 831.45 | 776.14 | 0.00 | 62.55 |
| VNNAVLFDK | 0 | 1018.54 | 358.92 | 357.71 | 66.69 |
|  |  |  | 1135.1 | 357.7 |  |
